# Monodisperse Fe_3_O_4_/SiO_2_ and Fe_3_O_4_/SiO_2_/PPy Core-Shell Composite Nanospheres for IBU Loading and Release

**DOI:** 10.3390/ma12050828

**Published:** 2019-03-11

**Authors:** Lazhen Shen, Bei Li, Yongsheng Qiao, Jinping Song

**Affiliations:** 1 School of Chemistry and Environmental Engineering, Institute of Applied Chemistry, Shanxi Datong University, Datong 037009, China; 13653621281@163.com (B.L.); songjphx@163.com (J.S.); 2 Department of Chemistry, Xinzhou Teachers University, Xinzhou 034000, China

**Keywords:** Fe_3_O_4_/SiO_2_, Fe_3_O_4_/SiO_2_/PPy, core-shell structure, magnetic property, drug loading, drug release, Korsmeyer-Peppas model

## Abstract

The magnetic targeting drug delivery system is an effective way of targeting therapy. In this study, the monodisperse Fe_3_O_4_ nanoparticles with a particles size of about 180 nm were first prepared via a solvothermal method. Subsequently, the core-shell structure Fe_3_O_4_/SiO_2_ and Fe_3_O_4_/SiO_2_/polypyrrole (PPy) composite nanospheres were successfully synthesized by coating Fe_3_O_4_ nanoparticles with SiO_2_ shell layer using the Stöber method and PPy shell by solvothermal method in turn. The as-prepared nanoparticles were characterized using transmission electron microscopy (TEM), X-ray diffraction (XRD), Fourier transform-infrared spectroscopy (FT-IR), vibrating sample magnetometer (VSM), thermogravimetric analysis (TGA), and Ultraviolet-Visible spectrophotometer (UV-Vis). The results indicated that the as-prepared composite nanospheres displayed a well-defined core-shell structure and monodispersity. The thicknesses of SiO_2_ shell and PPy shell were ~6 nm and ~19 nm, respectively. Additionally, the as-prepared nanoparticles exhibited high saturation magnetization of 104 emu/g, 77 emu/g, and 24 emu/g, and have great potential applications in drug delivery. The drug loading and drug release of the Fe_3_O_4_/SiO_2_ and Fe_3_O_4_/SiO_2_/PPy composite nanospheres to ibuprofen (IBU) under stirring and ultrasonication were investigated. Their drug loading efficiency and drug release efficiency under ultrasonication were all higher than 33% and 90%, respectively. The drug release analyses showed sustained release of IBU from nanospheres and followed the Korsmeyer-Peppas model.

## 1. Introduction

Ibuprofen (IBU) is the most commonly used and most frequently prescribed non-steroidal anti-inflammatory drug to relieve inflammation, fever, and pain from headache, migraine, toothache, joint pain, back pain, muscle pain, and menstrual cramps through oral administration [1,2,3,4]. However, its biological half-life is very short, resulting in frequent administration of drugs, which increases the toxic side effects of the system. Further, due to the extremely poor water solubility, oral administration can stimulate the gastrointestinal tract, produce side effects such as chest pain, headache, and vomiting, and in severe cases, can cause gastric bleeding, which limits the maximum daily dosage. To overcome these shortcomings, the drug delivery system can be used to deliver IBU directly to the infected site through parenteral route [5,6], and as the molecular size of IBU is only 0.6–1 nm, which is suitable to be connected to the nano-drug carrier [7,8,9], this makes it an ideal candidate for use in controlled release drug delivery systems.

At present, various composite nanoparticles, such as silica/mesoporous silica nanoparticles, metal-organic matrix nanocomposites, titanium dioxide nanoparticles, polymer nanofilms, and other nanomaterials [7,9,10,11,12,13,14,15,16,17,18,19,20,21], have been used for the loading and release of ibuprofen. Silica/mesoporous silica nanoparticles are non-toxic, biocompatible, biologically inert, and have strong stability even at 1000 °C [10,11,12,13,14]. A large number of hydroxyl groups on their surface can provide connection sites for drug loading to achieve drug delivery. A metal-organic framework analog to MIL-101(Cr) containing amine groups was synthesized by Silva et al. [15]. It was first found that the amino groups have a significant influence on the loading and release of IBU and nimesulide (NMS) due to the possibility of interactions between the functional groups in IBU and the NH_2_ groups in the as-prepared matrix. Pawlik et al. applied a co-delivery of gentamicin and ibuprofen from nanoporous anodic titanium dioxide layers and examined the effects of crystalline structure and thickness of the nanoporous TiO_2_ layer on the amount of released drugs and drug release profiles [16]. Mucoadhesive IBU-loaded chitosan films suitable for oral mucosal drug delivery were prepared, which is an alternative green process in the drug delivery system [7]. Although these nano-drug carriers can achieve high-efficiency loading of IBU and slow controlled release in vitro, they are still lacking targeting in vivo and cannot directly target the disease center that produces certain toxic side effects. Therefore, it is necessary to combine these materials with magnetic nanoparticles to obtain magnetic targeting drug carriers to directly target IBU to the disease center under the action of an external magnetic field, minimizing the toxicity and side effects of the system.

Fe_3_O_4_ magnetic nanoparticles show good application prospects in the targeting drug delivery system (TDDS) due to their high magnetism, good stability, and biocompatibility [22,23]. The solvothermal method is a commonly used method to prepare Fe_3_O_4_ magnetic nanoparticles. A new core-shell microsphere Fe_3_O_4_@MOFs/GO was synthesized by exploring the layer-by-layer self-assembly method for IBU drug loading properties [24]. However, the size of the microspheres prepared exceeds 400 nm and is not suitable for targeting drug delivery in vivo—in order for drug carriers to successfully pass through the 100–1000 nm gap between neovascularization cells such as alveoli and capillaries without causing embolism, the particle size threshold of drug carriers in tumor exosmosis should be about 400 nm, preferably below 200 nm [25]. The Fe_3_O_4_ microspheres with diameters of 400–800 nm obtained using the solvothermal method were more easily swallowed by the reticuloendothelial system and could not achieve drug delivery [26]. Therefore, controlling the size of Fe_3_O_4_ magnetic nanoparticles through the solvothermal method is still a major challenge. Polypyrrole (PPy) has excellent electrical conductivity, stability, biocompatibility, and biodegradability, and the amino groups on the skeleton can provide the connection sites for the drug and connect hydrophobic drugs through π–π stacking action [27,28,29].

In this paper, Fe_3_O_4_ nanospheres with a size of about 180 nm were obtained by the solvothermal method using ethylene glycol as a reductant and solvent and polyethylene glycol as a surfactant. Subsequently, the core-shell structure Fe_3_O_4_/SiO_2_ and Fe_3_O_4_/SiO_2_/PPy composite nanospheres were synthesized by combining Fe_3_O_4_ nanoparticles with SiO_2_ and PPy. The drug controlled release behavior of these two composite nanospheres to IBU under stirring and ultrasonication was studied. Fe_3_O_4_ nanoparticles can endow composite nanospheres with magnetic field-mediated targeting drug delivery capability. Moreover, SiO_2_ and PPy can not only prevent magnetic Fe_3_O_4_ core from aggregating, but also have non-toxicity, stability, biocompatibility, and biodegradability, which can provide more connected sites for IBU drug load and can be efficiently located at the tumor site to achieve targeted drug delivery.

## 2. Materials and Methods

### 2.1. Preparation of Fe_3_O_4_ Nanoparticles

The Fe_3_O_4_ nanoparticles were prepared via a solvothermal method. Typically, 6.75 g of FeCl_3_·6H_2_O was dissolved in ethylene glycol to obtain an orange-yellow solution. When the FeCl_3_·6H_2_O was completely dissolved, 1.0–1.5 g of the surfactant polyethylenegl-4000 (PEG-4000) was added, and 11–12 g of anhydrous sodium acetate was added after the dissolving of PEG-4000. After continuous stirring for 30 min, the mixed solution was transferred into a Teflon-sealed autoclave at 200 °C for 6–8 hours. After the autoclave was cooled to room temperature, the black products were washed alternately with distilled water and anhydrous ethanol several times. The obtained Fe_3_O_4_ nanoparticles were ultrasonically dispersed in distilled water for further reaction. Among them, ethylene glycol was used as a reductant and solvent, sodium acetate was hydrolyzed to produce hydroxyl groups to precipitate iron ions and ferrous ions, and polyethylene glycol was used as a surfactant to improve the dispersion of particles.

### 2.2. Preparation of Fe_3_O_4_/SiO_2_ Composite Nanospheres

The SiO_2_ shell modification was carried out on the obtained Fe_3_O_4_ nanoparticles by the Stöber method [30,31]. Firstly, 50 mL of absolute ethanol, 1 mL of deionized water, 2 mL of ammonium hydroxide (25%), and 300 mL of tetraethyl orthosilicate (TEOS) were mixed and reacted in a water bath at 40 °C for 10 min. The TEOS was hydrolyzed to obtain primary SiO_2_ nanoparticles, and then the ultrasonically dispersed Fe_3_O_4_ nanoparticles were added to the above mixture. After mechanically stirring the mixture for 12 h at room temperature, the products were magnetically separated and washed several times with distilled water and absolute ethanol, respectively. Finally, the obtained Fe_3_O_4_/SiO_2_ composite nanospheres were dried or re-dispersed in distilled water by ultrasound for further coating of the polypyrrole shell.

### 2.3. Preparation of Fe_3_O_4_/SiO_2_/PPy Composite Nanospheres

Polypyrrole shell layer was prepared by the hydrothermal method [32]. In general, the pyrrole monomer (distilled before reaction and stored in a brown bottle in a refrigerator at 4 °C) was added dropwise to the above Fe_3_O_4_/SiO_2_ composite microspheres solution under stirring with the ratio of Fe_3_O_4_/SiO_2_ composite microspheres to pyrrole monomer of 100–200 mg:300 μL. After mechanical stirring for 1 h, this mixture was transferred into a Teflon-sealed autoclave, and the ammonium persulfate solution was added drop by drop (0.1–0.2 g ammonium persulfate dissolved in 10 mL distilled water). After 8 h of reaction at 140 °C, the autoclave was cooled to room temperature, and then the precipitate was separated by a magnet and washed with distilled water and anhydrous ethanol for several times. Finally, the Fe_3_O_4_/SiO_2_/PPy composite nanospheres were obtained by drying at room temperature.

### 2.4. Characterizations

The morphology and size of the samples were characterized by BDX-3300 JEOL 100CX-II transmission electron microscope (JEOL, Akishima, Tokyo). The X-ray diffraction was obtained by Bruker D8 Focus diffractometer (Bruker, Billerica, MA, USA) to analyze the crystalline phase of the products. Fourier-transform infrared spectroscopy was conducted on an FTIR-650 spectrometer to determine the composition and structure of the particles (Gangdong Sci. & Tech. Development Co., Ltd., Tianjin, China). The magnetic properties of the particles were studied by MPMS SQUID vibrating sample magnetometer (VSM, Quantum Design Co., Ltd., San Diego, CA, USA). Thermogravimetric analyses and differential thermogravimetric analyses (DTG, NETZSCH-Gerätebau GmbH, Selb, Germany) were performed using the TG 209 F3 Tarsus instrument under an air atmosphere with a heating rate of 10 °C/min from room temperature up to 1000 °C. The concentration of IBU in the supernatant was measured by an alpha-1860Plus ultraviolet-visible spectrophotometer (LSI, Shanghai, China).

### 2.5. Drug Loading and Drug Release

The drug loading and drug release of the prepared nanospheres for IBU were performed in 0.2 mol/L of disodium hydrogen phosphate–sodium dihydrogen phosphate buffer solution (PB) at the physiological conditions of a temperature of 37 °C and a pH of 7.4 [33,34]. The content of IBU in buffer solution was quantified using UV-Vis absorption technique at λ_max_ = 264 nm. A total of 10 mg of Fe_3_O_4_/SiO_2_ and Fe_3_O_4_/SiO_2_/PPy core-shell composite nanospheres were added in 25 mL of 1 mg/mL IBU-ethanol solution, respectively. After stirring for 24 h, the supernatant was immersed and washed with anhydrous ethanol, and then the absorbance of supernatant and cleaning solution was measured by UV-Vis spectrophotometer to calculate the residual unloaded IBU drug concentration, thereby indirectly obtaining the drug loading of the two nanospheres. The IBU drug loading (*DL*, mg) is defined as Equation (1):

(1) $$DL=C_{0}V_{0}-C_{1}V_{1}-C_{2}V_{2}$$

where *C*_0_, *C*_1_, and *C*_2_ are the initial concentration of the drug and the concentration of the supernatant and the cleaning solution, respectively, in mg/mL. *V*_0_, *V*_1_, and *V*_2_ are the initial volume of the drug and the volume of supernatant and cleaning solution, respectively, in mL.

The drug loading efficiency (*E*_a_, %) is calculated according to Equation (2):

(2) $$E_{a}=\frac{DL}{m}\times 100$$

where *m* is the amount of as-prepared composite nanospheres added, in mg.

The calculation formula of encapsulation efficiency (*EE*, %) is as Equation (3) [2]:

(3) $$EE=\frac{DL}{m_{\mathrm{IBU}}}\times 100$$

where *m*_IBU_ is the amount of loaded IBU, in mg.

For in vitro drug release, these two IBU-loaded composite nanospheres were put into dialysis bags separately and incubated in 25 mL of 0.2 mol/L PB buffer solution (pH = 7.4), and 5 mL of the supernatant solution was removed after mechanical stirring for 0.5 h, 1 h, 1.5 h, 2 h, 2.5 h, 3.5 h, 4 h, 5 h, 6 h, 7 h, 8 h, 9 h, 10 h, 11 h, 24 h, and 84 h, respectively. Drug concentration in the supernatant solution was determined as before, and the precipitate was re-suspended with the equivolumetric fresh PB buffer solution with the same pH and concentration. The drug release efficiency (*DR*, %) is defined as Equation (4) [9]:

(4) $$DR=\frac{m_{R}}{m_{\mathrm{IBU}}}\times 100$$

where *m*_R_ is the amount of released IBU, in mg.

In order to study the drug release behavior under ultrasound, the two IBU-loaded composite nanospheres were put in 25 mL of 0.2 mol/L, pH = 7.4 PB buffer solution. Under ultrasound, 5 mL of supernatant was removed after 5 min, 10 min, 15 min, 20 min, 30 min, 40 min, 50 min, 60 min, and 65 min, respectively, and replaced with the equivolumetric fresh pH = 7.4 PB buffer solution. The absorbance of the supernatant was measured, and the drug release curves of the two drug-loaded nanoparticles under ultrasound were obtained.

To describe the release profile, the common exponential equation of the Korsmeyer-Peppas model was used. The Korsmeyer-Peppas model [35,36,37] derived a simple mathematical relationship which described the drug release from the above two systems, and can be defined as Equation (5):

(5) $$W=\frac{M_{t}}{M_{\infty }}=kt^{n}$$

where *W* is the fraction of drug released at time *t*, *M*_t_ is the cumulative released amount at time *t*, *M*_∞_ is the total released amount, *t* is the time, *k* is the kinetic constant, and *n* is the release exponent.

## 3. Results and Discussion

**Figure 1:**
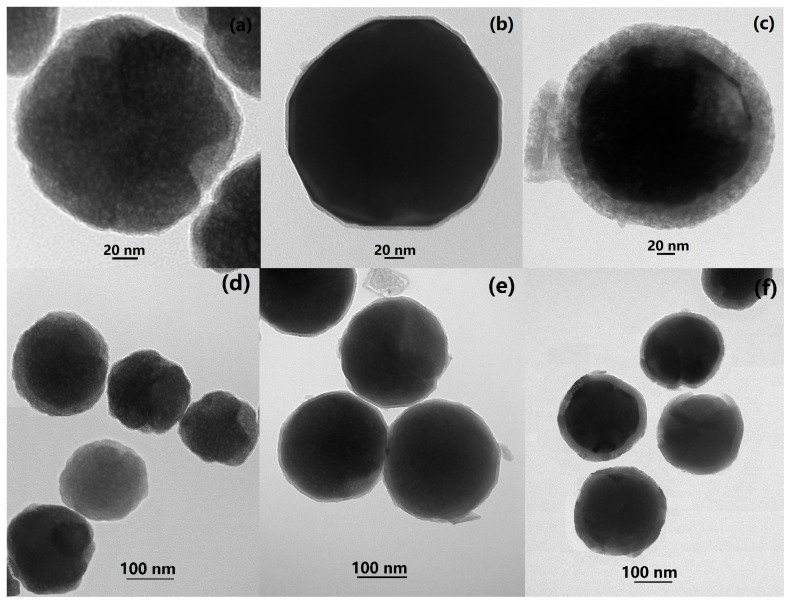
TEM images of (**a**,**d**) Fe_3_O_4_ nanoparticles, (**b**,**e**) Fe_3_O_4_/SiO_2_ core-shell composite nanospheres, and (**c**,**f**) Fe_3_O_4_/SiO_2_/PPy core-shell composite nanospheres.

Figure 1 gives the TEM images of the synthesized Fe_3_O_4_ nanoparticles, Fe_3_O_4_/SiO_2_ core-shell composite nanospheres, and Fe_3_O_4_/SiO_2_/PPy core-shell composite nanospheres. It can be seen that the Fe_3_O_4_ had spherical nanoparticles with an average size of about 180 nm and was monodispersed (Figure 1a,d). From Figure 1b,e, it is found that the SiO_2_ layer had deposited onto the surface of Fe_3_O_4_ nanoparticles and there was an interface which can be clearly distinguished between the inner Fe_3_O_4_ magnetic core and the outer SiO_2_ shell. The thickness of the SiO_2_ shell was about 6 nm, which can enhance the stability and biocompatibility of Fe_3_O_4_ nanoparticles. According to Figure 1c,f, the PPy also had uniformly deposited on the surface of Fe_3_O_4_/SiO_2_ composite nanospheres and formed a PPy shell of about 19 nm. After the coating process of SiO_2_ and PPy, the average diameters of the Fe_3_O_4_/SiO_2_ composite nanospheres and the Fe_3_O_4_/SiO_2_/PPy composite nanospheres increased to around 192 nm and 230 nm, respectively. It can be clearly observed that both the obtained Fe_3_O_4_/SiO_2_ composite nanospheres and Fe_3_O_4_/SiO_2_/PPy composite nanospheres had obvious core-shell structures and were monodispersed.

**Figure 2:**
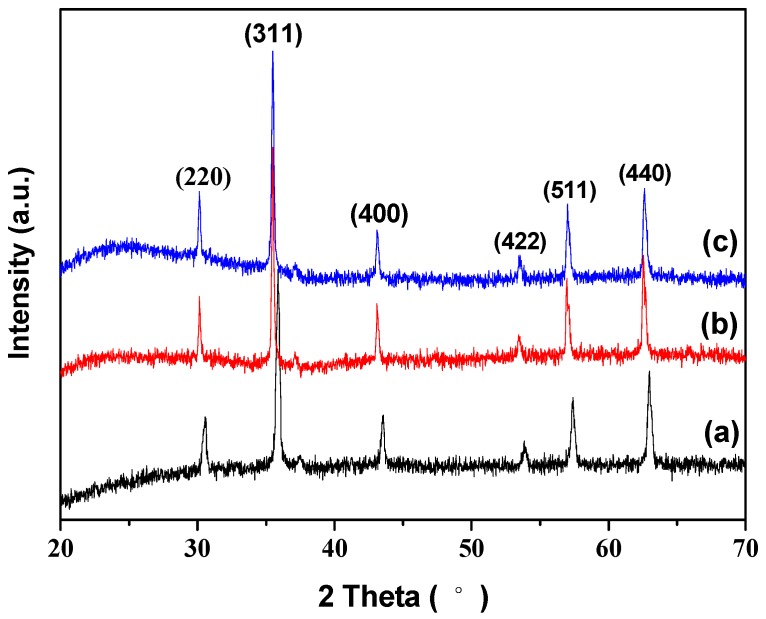
XRD patterns of (a) Fe_3_O_4_ nanoparticles, (b) Fe_3_O_4_/SiO_2_ core-shell composite nanospheres, and (c) Fe_3_O_4_/SiO_2_/PPy core-shell composite nanospheres.

The XRD patterns of Fe_3_O_4_ nanoparticles, Fe_3_O_4_/SiO_2_ core-shell composite nanospheres, and Fe_3_O_4_/SiO_2_/PPy core-shell composite nanospheres are illustrated in Figure 2. Six characteristic diffraction peaks at 2θ = 30.53°, 35.86°, 43.45°, 53.78°, 57.34°, and 62.96°, corresponding to (220), (311), (400), (422), (511), and (440) diffraction planes, respectively, were observed for all samples, which are consistent with the database of magnetite diffraction card (JCPDS 19-0629) [38,39]. This indicates that the magnetite crystalline phase remained in the two composite nanospheres. A weak broad peak at 2θ = 22° in Figure 2b was assigned to the amorphous SiO_2_, indicating the formation of Fe_3_O_4_/SiO_2_ core-shell composite nanospheres [40]. Due to the contribution of PPy, the strength of the broad peak between 21° and 28° in Figure 2c increased compared with Figure 2b, indicating that the Fe_3_O_4_/SiO_2_/PPy core-shell composite nanospheres were synthesized [41].

**Figure 3:**
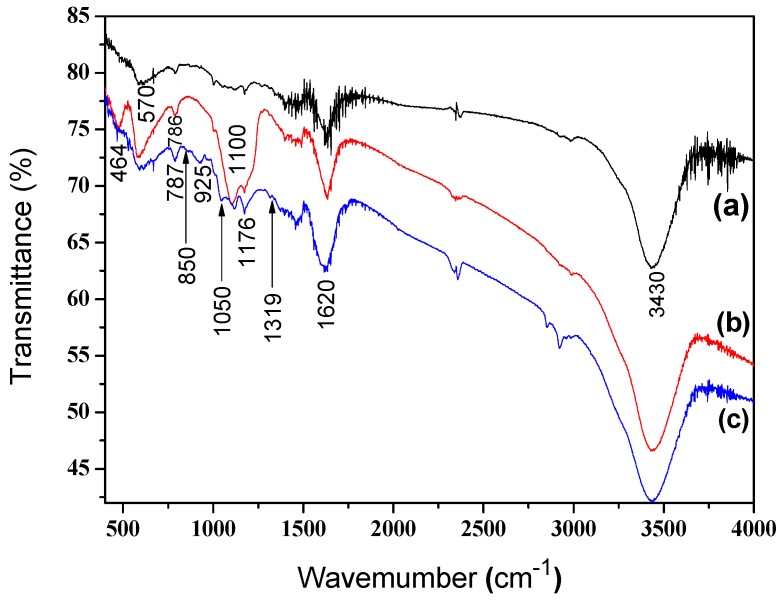
FT-IR spectra of (a) Fe_3_O_4_ nanoparticles, (b) Fe_3_O_4_/SiO_2_ core-shell composite nanospheres, and (c) Fe_3_O_4_/SiO_2_/PPy core-shell composite nanospheres.

The FT-IR analysis is further employed to characterize the functional groups and structures of the Fe_3_O_4_ nanoparticles, Fe_3_O_4_/SiO_2_ core-shell composite nanospheres, and Fe_3_O_4_/SiO_2_/PPy core-shell composite nanospheres, and the results are presented in Figure 3. The characteristic peaks of Fe–O stretching vibration for the three particles were all observed around 570 cm^−1^ [42,43]. Three peaks at about 464 cm^−1^, 786 cm^−1^, and 1100 cm^−1^ in Figure 3b,c were attributed to the tensile vibration of Si–O, Si–O–Si, and Si–OH bonds, respectively, indicating the presence of SiO_2_ in the Fe_3_O_4_/SiO_2_ core-shell composite nanospheres [44,45,46]. The peak in Figure 3c was shifted from 786 cm^−1^ in Figure 3b to 787 cm^−1^ due to the contribution of C–H out-plane ring bend at 790 cm^−1^ in polypyrrole. The peaks at 850 cm^−1^, 925 cm^−1^, and 1176 cm^−1^ in Figure 3c were assigned to the C–H in-plane/out-plane deformation vibration of PPy [42,47]. Bands at 1050 cm^−1^ and 1319 cm^−1^ observed in Figure 3c can be attributed to C–H in-plane vibration and C–N stretching vibration of PPy, respectively [48]. The characteristic peaks at 1602 cm^−1^ and 3430 cm^−1^ observed in all samples corresponded to the stretching vibration of the O–H bond, which indicates that the adsorbed OH^−^ groups exist in the samples [44]. Compared with Fe_3_O_4_ nanoparticles and Fe_3_O_4_/SiO_2_ composite nanospheres, there were sharper characteristic peaks at 1602 cm^−1^ and 3430 cm^−1^ in Fe_3_O_4_/SiO_2_/PPy composite nanospheres due to the C=C and N–H stretching vibration absorption bands of polypyrrole at these two peaks [1], indicating the formation of Fe_3_O_4_/SiO_2_/PPy composite nanospheres.

**Figure 4:**
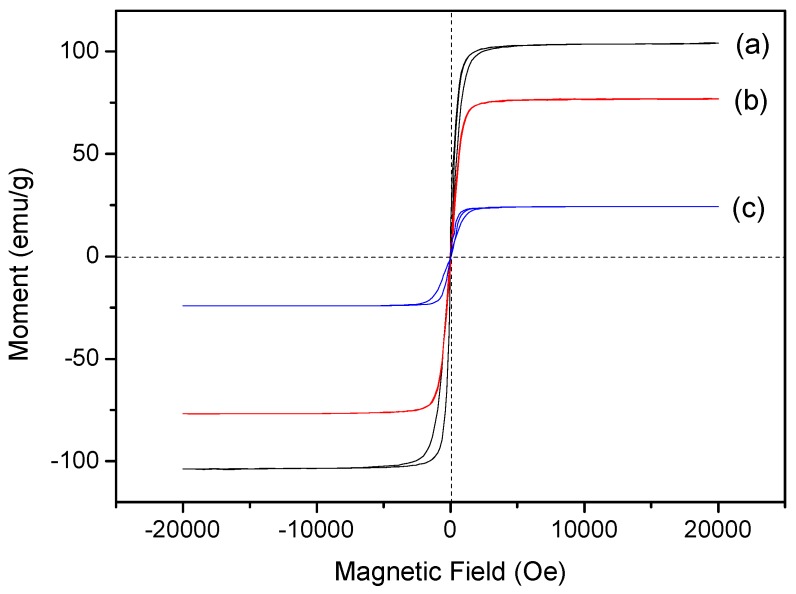
Room temperature magnetization curves of (a) Fe_3_O_4_ nanoparticles, (b) Fe_3_O_4_/SiO_2_ core-shell composite nanospheres, and (c) Fe_3_O_4_/SiO_2_/PPy core-shell composite nanospheres.

The diagram of magnetization vs. magnetic field at room temperature for Fe_3_O_4_ nanoparticles, Fe_3_O_4_/SiO_2_ core-shell composite nanospheres, and Fe_3_O_4_/SiO_2_/PPy core-shell composite nanospheres are depicted in Figure 4. The Fe_3_O_4_ nanoparticles exhibited a high saturation magnetization of 104 emu/g as shown in Figure 4a. After coated with the SiO_2_ shell, the saturation magnetization of Fe_3_O_4_/SiO_2_ core-shell composite decreased to 77 emu/g [49], indicating that the SiO_2_ layer was successfully connected to the magnetic core. The saturation magnetization of Fe_3_O_4_/SiO_2_/PPy core-shell composite nanospheres was further reduced to 24 emu/g, which indicates the successful modification of PPy shell on the SiO_2_ shell [50]. The saturation magnetization of the latter had a significant decrease, further confirming that the thickness of the PPy shell was thicker than that of the SiO_2_ layer, which is consistent with TEM results that the SiO_2_ layer was 6 nm and the PPy shell was 19 nm. Even if the saturation magnetization of the final Fe_3_O_4_/SiO_2_/PPy core-shell composite nanospheres decreases, the magnetic nanospheres can still be triggered to release targeting drugs under the external magnetic field. At the same time, the magnetic nanospheres can be stratified immediately after ultrasonic dispersion and are suitable for drug release research under ultrasound triggering. The values of remnant magnetization of Fe_3_O_4_ nanoparticles, Fe_3_O_4_/SiO_2_, and Fe_3_O_4_/SiO_2_/PPy composite nanospheres are 4.10 emu/g, 4.09 emu/g, and 1.50 emu/g, respectively, and their coercivities are all zero Oe. It can be seen that all the prepared samples have good magnetic properties, indicating that they apply to targeted drug/gene delivery under the action of an external magnetic field.

**Figure 5:**
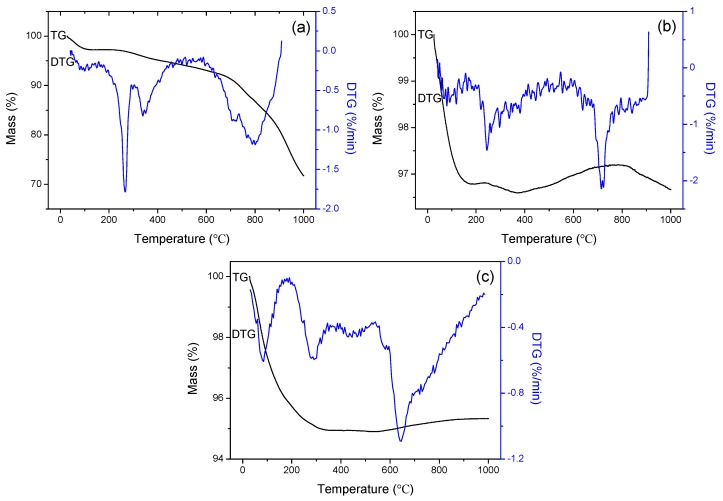
TGA and differential thermogravimetric analysis (DTG) curves of (**a**) Fe_3_O_4_ nanoparticles, (**b**) Fe_3_O_4_/SiO_2_ core-shell composite nanospheres, and (**c**) Fe_3_O_4_/SiO_2_/PPy core-shell composite nanospheres.

The thermal stability of Fe_3_O_4_ nanoparticles, Fe_3_O_4_/SiO_2_ core-shell composite nanospheres, and Fe_3_O_4_/SiO_2_/PPy core-shell composite nanospheres was further studied by thermogravimetric analysis and differential thermogravimetric analysis, and their TGA and DTG curves are illustrated in Figure 5. For Fe_3_O_4_ nanoparticles, there are four stages of mass loss and two weight gain steps as shown in Figure 5a. The first stage of about 3% mass loss from room temperature to 110 °C is due to the evaporation of adsorbed water, corresponding to the endothermic peak at about 100 °C. The weight loss during 230–700 °C can be attributed to the decomposition of crystalline water and the adhered hydroxyl from a small amount of ethylene glycol solvent, polyethylene glycol surfactant, and adsorbed hydroxyl at the second stage [51]. At the second and the third endothermic peaks at 266 °C and 338 °C, they are assigned to the loss of the crystal water, structure water within Fe_3_O_4_ crystal, and the loss of the adhered hydroxyl. The sharp endothermic peak at 266 °C corresponds to the formation of γ-Fe_2_O_3_ with spinel phase from Fe_3_O_4_. The weight loss at 700–1000 °C may be ascribed to the phase transition of Fe_3_O_4_ nanoparticles [52]. Two obvious endothermic peaks at 710 °C and 789 °C resulting from the disappearance of spinel crystal were observed because of the change from phase γ-Fe_2_O_3_ to α-Fe_2_O_3_ phase (Curie temperature of α-Fe_2_O_3_ T_c_ = 747 °C). For Fe_3_O_4_/SiO_2_ core-shell composite nanospheres (Figure 5b), only about 3.4% of the weight loss was caused, indicating that the thermally stable SiO_2_ shell was successfully deposited onto the surface of magnetic core [53], and the loss mainly between room temperature and 160 °C, corresponding to the endothermic peak at 100 °C, might be owing to the decomposition of the adsorbed water and ethanol. The endothermic peak at 710 °C may be the volatile removal of high temperatures. From Figure 5c, 5% weight loss in the Fe_3_O_4_/SiO_2_/PPy composite nanospheres during room temperature and 300 °C was associated with the evaporation of absorbed water and removal of pyrrole monomer, in keeping with the two endothermic peaks at 98 °C and 290 °C. There was a weak peak at 400–500 °C and a sharp peak at 638 °C due to the destruction and decomposition of PPy chains, which indicates that PPy chains can only be destroyed at a higher temperature and have good stability. There is no mass loss at around 400 °C, indicating that there were no polypyrrole chains that were degraded. The higher mass loss in Figure 5c than Fe_3_O_4_/SiO_2_ composite nanospheres may be related to the thermal instability of the coated PPy shell on the SiO_2_ layer [54]. These results indicate that the SiO_2_ shell layer and the PPy shell are present in Fe_3_O_4_/SiO_2_/PPy composite nanospheres.

Table 1 lists the drug loading, encapsulation efficiency, and drug loading efficiency of the Fe_3_O_4_/SiO_2_ and Fe_3_O_4_/SiO_2_/PPy core-shell composite nanospheres for the same concentration of IBU-ethanol solution, and lists the drug release efficiency of the two IBU-loaded nanospheres under stirring and ultrasound, respectively. The Fe_3_O_4_/SiO_2_/PPy composite nanospheres exhibited higher drug loading, encapsulation efficiency, and drug loading efficiency than that of the Fe_3_O_4_/SiO_2_ composite nanospheres. The reason is that in Fe_3_O_4_/SiO_2_/PPy composite nanospheres, not only SiO_2_ can provide binding sites for IBU, but also the modified PPy outer layer with a large specific surface area and the amino groups on the framework of PPy can make drugs bond with nanospheres through hydrogen bonds as well as π–π stacking, which increases the drug loading capacities of the double-shell composite nanospheres.

**Figure 6:**
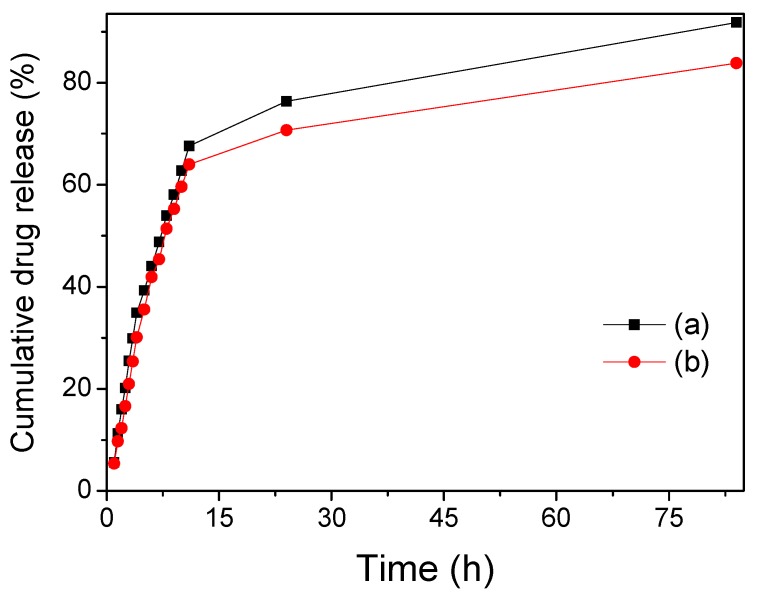
Drug release curves of (a) Fe_3_O_4_/SiO_2_ core-shell composite nanospheres and (b) Fe_3_O_4_/SiO_2_/PPy core-shell composite nanospheres under stirring.

In order to investigate the effect of drug release behavior, the Fe_3_O_4_/SiO_2_ and Fe_3_O_4_/SiO_2_/PPy core-shell composite nanospheres were loaded by IBU-ethanol solution with the same concentration and then released under the same conditions. The drug release profiles of IBU from IBU-loaded composite nanospheres under stirring are graphically represented in Figure 6. It can be seen that these two composite nanospheres showed similar drug release behavior, except that the Fe_3_O_4_/SiO_2_ core-shell composite nanospheres exhibited a slightly faster rate and a slightly higher release efficiency. With the increase of shell layer, the drug release rate slowed down, because, in the double-shell composite nanospheres, the drug release not only passes through the middle shell, but also through the outer shell, and their presence slows down the drug release rate. Similar to the study from Prabha et al., their team has synthesized and characterized Fe_3_O_4_-cyclodextrin (CD), Fe_3_O_4_-CD-polyethyleneglycol (PEG) and Fe_3_O_4_-CD-PEG-polyethyleneimine (PEI) nanocomposites loaded with 5-Fu. The results show that the electrostatic interaction and hydrogen bond interaction between the nanocomposites and drug molecules increased with the increase of the coatings, and then the drug encapsulation efficiency and drug loading efficiency increase. Among them, Fe_3_O_4_-CD-PEG-PEI nanocomposites are more suitable as the drug carriers. Similarly, with the increase of polymer coatings, drug release time increased, and slow drug release can be achieved. Therefore, the prepared Fe_3_O_4_-CD-PEG-PEI nanocomposites can be better used for anti-cancer drug delivery in cancer therapy [55]. From Figure 6, all of the nanospheres released about >35% of IBU in the first 5 h and >65% after 12 h. Most of the drugs adsorbed in the outer surface layer can be released, as well as some drugs bonded by hydrogen bonds that were also released continuously in 12 h. Other drugs attached to the inside of the composite nanospheres further reduce the release rate and achieve a slow drug release and meet the long-term continuous drug use of the human body. After 84 hours of drug release, the Fe_3_O_4_/SiO_2_ and Fe_3_O_4_/SiO_2_/PPy composite nanospheres reached an efficient drug release rates of 91.82% and 83.86%, respectively, indicating the obtained composite nanospheres can be used as a better magnetic targeting drug delivery system.

**Figure 7:**
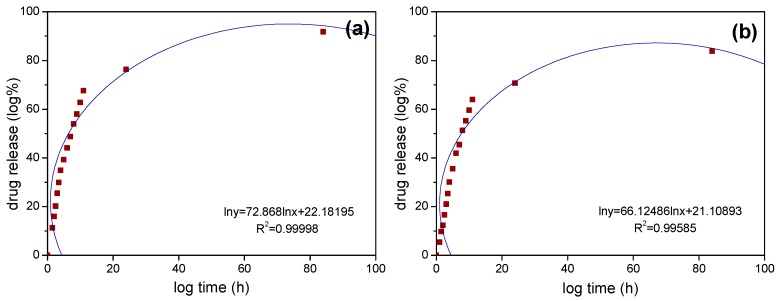
Drug release kinetic curves with Korsmeyer-Peppas model of (**a**) Fe_3_O_4_/SiO_2_ core-shell composite nanospheres and (**b**) Fe_3_O_4_/SiO_2_/PPy core-shell composite nanospheres under stirring.

Figure 7 shows the kinetic curves of Korsmeyer-Peppas model of Fe_3_O_4_/SiO_2_ and Fe_3_O_4_/SiO_2_/PPy core-shell composite nanospheres under stirring to describe the mechanism of sustained release kinetics. Further, the kinetic model parameters, including R-square values, kinetic constant, and release exponent, are given in Table 2. From Figure 7, we can see that there are good linear relationships between the logarithm of cumulative release and logarithm of time in the whole process of IBU release from the two nanospheres. Table 2 shows that the release exponents of the Korsmeyer-Peppas model are 0.3409 and 0.3606, respectively, and less than 0.5, therefore indicating that the release kinetics of the sustained release system conforms to Fick diffusion mechanism [56,57].

**Figure 8:**
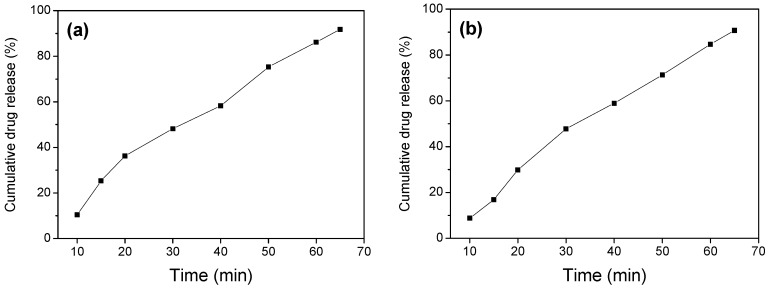
Drug release curves of (**a**) Fe_3_O_4_/SiO_2_ core-shell composite nanospheres and (**b**) Fe_3_O_4_/SiO_2_/PPy core-shell composite nanospheres under ultrasonication.

Because both Fe_3_O_4_/SiO_2_ and Fe_3_O_4_/SiO_2_/PPy composite nanospheres can be separated quickly after ultrasonication, the study of the drug release behavior under ultrasonication of the two composite nanospheres as shown in Figure 8 can be compared with that under stirring [58]. As can be seen from Figure 8, the drug release efficiency under ultrasonication was significantly accelerated, and the drug release efficiency reached about 90% of IBU within 65 min. This is due to the fact that the drug release mechanism no longer follows the 0-level release mode under the action of ultrasound, making the drug release efficiency significantly faster than that under stirring, which is consistent with the research by Shamsipur et al. [59]. The chitosan (CS)-Fe_3_O_4_ nanoparticles were synthesized and connected with ciprofloxacin through a hydrogen bond. The drug loading and the controlled release behavior of ciprofloxacin loaded CS-Fe_3_O_4_ under ultrasound was studied. The drug loading of ciprofloxacin reached 99%. Without ultrasound, the drug was sustainably released in the first 400 min, and the release was slow after five days. When low-frequency ultrasound was used, the drug release was promoted, and 95% of the drug was released within 60 min, achieving controlled release of the drug. These results indicate that the obtained Fe_3_O_4_/SiO_2_ and Fe_3_O_4_/SiO_2_/PPy composite nanospheres can not only be used for controlled drug release under stirring, but also provide a new drug carrier for drug release under the action of ultrasound.

**Figure 9:**
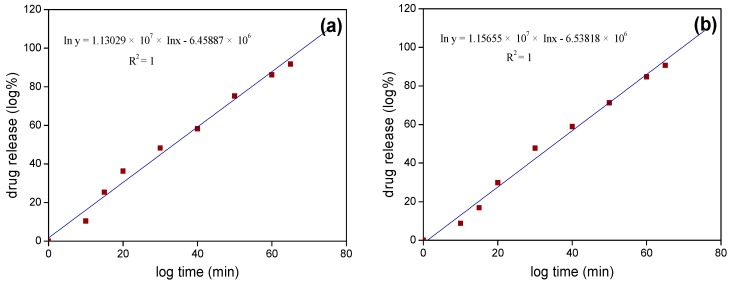
Drug release kinetic curves with Korsmeyer-Peppas model of (**a**) Fe_3_O_4_/SiO_2_ core-shell composite nanospheres and (**b**) Fe_3_O_4_/SiO_2_/PPy core-shell composite nanospheres under ultrasonication.

The sustained release kinetic curves and the kinetic model parameters of Fe_3_O_4_/SiO_2_ and Fe_3_O_4_/SiO_2_/PPy core-shell composite nanospheres under ultrasonication are shown in Figure 9 and Table 3. It can be seen that the two release systems had the release exponent value *n* = 0.9008 and 0.9907, respectively. The magnitude of release exponent indicates that the kinetic release mechanism was non-Fick diffusion [36].

## 4. Conclusions

In summary, we report a monodisperse Fe_3_O_4_ nanoparticle with a particle size of about 180 nm obtained via a solvothermal method. The facile Stöber and hydrothermal methods were utilized to prepare the Fe_3_O_4_/SiO_2_ and Fe_3_O_4_/SiO_2_/PPy composite nanospheres with obvious core-shell structure and monodispersity by coating Fe_3_O_4_ nanoparticles with SiO_2_ and PPy shell in turn. The as-prepared Fe_3_O_4_ nanoparticles, and the Fe_3_O_4_/SiO_2_ and Fe_3_O_4_/SiO_2_/PPy composite nanospheres displayed the high saturation magnetizations of 104 emu/g, 77 emu/g, and 24 emu/g, respectively, indicating the great potential applications in drug delivery. The drug loading efficiency and drug release efficiency of the two composite nanospheres were all higher than 33% and 90% under ultrasonication, respectively. The release process of nanospheres under stirring followed a Fick diffusion mechanism, while nanospheres under ultrasonication followed a non-Fick diffusion. Therefore, this study may provide new functional nanocomposites for IBU loading and release.

## Figures and Tables

**Table 1:** Ibuprofen (IBU) loading and release of Fe_3_O_4_/SiO_2_ and Fe_3_O_4_/SiO_2_/PPy core-shell composite nanospheres.

Sample	Fe_3_O_4_/SiO_2_	Fe_3_O_4_/SiO_2_/PPy
Concentration of IBU (mg/mL)	1	1
Drug loading (mg)	3.329	3.640
Encapsulation efficiency (%)	13.32	14.56
Drug loading efficiency (%)	33.29	36.40
Drug release efficiency (%)	91.82 (stirring for 84 h)	83.86 (stirring for 84 h)
	91.82 (ultrasound for 65 min)	90.73 (ultrasound for 65 min)

**Table 2:** Parameters of Korsmeyer-Peppas model for IBU release under stirring.

Sample	R^2^	*k*	*n*
Fe_3_O_4_/SiO_2_	0.99998	22.36	0.3409
Fe_3_O_4_/SiO_2_/PPy	0.99585	19.89	0.3606

**Table 3:** Parameters of Korsmeyer-Peppas model for IBU release under ultrasonication.

Sample	R^2^	*k*	*n*
Fe_3_O_4_/SiO_2_	1	2.165	0.9008
Fe_3_O_4_/SiO_2_/PPy	1	1.398	0.9907
